# Volume Resistivity of Viton Polymer under Thermal Aging

**DOI:** 10.3390/polym13050773

**Published:** 2021-03-03

**Authors:** Alireza Abdihamzehkolaei, Md Tanvir Ahad, Zahed Siddique

**Affiliations:** School of Aerospace and Mechanical Engineering, University of Oklahoma, Norman, OK 73019, USA; Md.Tanvir.Ahad-1@ou.edu

**Keywords:** volume resistivity, viton, insulating materials, polymers, nanocomposites, aging

## Abstract

This study examines the influence of various electrical parameters on the volume resistivity of the Viton fluoroelastomer. The transient current, the temperature dependence of volume resistivity, the voltage dependence of resistivity, and the surface morphology of Viton insulators are investigated for new and aged specimens. An accelerated aging process has been employed in order to simulate the natural aging of insulators in service. A detailed comparison between the new and aged samples is presented. The transient effect, which is a challenge to the resistivity measurement of insulators, has been investigated. The first 60 s of the resistivity measurement test showed a significant influence from the transient effect and should be excluded from the data. The volume resistivity of both new and aged samples decreased when the temperature increased. However, the resistivity of the aged sample was lower than the new one at all tested temperatures. When the temperature increased from 35 to 190 °C, resistivity decreased from 4.77 × 10^10^ to 6.99 × 10^8^ Ω-cm for the new sample and from 2.6 × 10^10^ to 6.68 × 10^8^ Ω-cm for the aged sample under 500 V. Additionally, the results from this study showed that the volume resistivity is inversely proportional to the applied voltage. Finally, scanning electron microscope (SEM) micrographs/images allowed us to closely examine the surface morphology of new and aged Viton samples. The surface of aged samples has been recognized with higher surface roughness and more significant surface cracks leading to poor performance under high voltage applications.

## 1. Introduction

In the 21st century, for outdoor insulation applications with the need for high-temperature resistance and strength, polymeric insulators have replaced ceramic materials. Polymers have proven to be desirable in high-temperature applications and are categorized as high-temperature resins [1]. Nanocomposites have aroused great interest in the scientific community due to the molecular-level reactions with the polymeric chain and high surface areas of nanofillers [2,3]. Many researchers have already devoted much effort during the last decade to understanding the effects of the inclusion of metallic fillers on the electrical properties of polymeric materials. Carbon black fillers are used with polymers to achieve higher electrical/thermal conductivity. However, the critical volumetric concentration of the polymeric materials fillers has been the key interest since resistivity decreases when the filler fractions increase [4,5,6]. The magnitude and temperature dependence of a polymer’s electrical resistivity is a function of its molecular structure, the nature and number of the current carriers, and the temperature [7,8]. Theoretical and experimental efforts have been continuously developing polymers filled with carbon black in the applications of discharging static electricity and electrical shielding. For various applications relying on the electrical resistivity at high temperatures, carbon-filled polymeric materials have a great interest in the scientific world [4,9,10].

In the offshore oil, gas, and energy automobile and aerospace industries, the hydraulic fracturing industry fluoroelastomers (Viton rubber or fluorocarbon (FKM) are widely used due to their excellent properties of resistance to heat, oil, chemicals, and solvents, their rigidity, and their thermal stability [11]. Viton rubber is mainly used in shaft seals, gaskets, O-rings, and fuel hoses for its excellent strength and low weight characteristics [12,13]. Fluoroelastomers such as Viton are used in synchrony with release agents and fusing oils [14,15,16]. Viton (dipolymers of hexafluoropropylene and vinylidene fluoride) is a fluorine-rich polymer with low melting point and plasticity, which imparts the mechanical and electrical integrity of the composites [17,18].

Bulk or volume resistivity, leakage current, and surface resistivity appear to be the main key properties of insulating fluoroelastomer materials. Still, the accurate measurement of these properties is reasonably complex since different factors have an impact on their values. Humidity, surface pollution, chemical reactions, temperature, impurities and additives, chemical structure, as well as time are the key factors that have effects on materials resistivity [19]. Efforts have been found in measurements of the electrical resistivity of various materials, but comparatively, very few accurate repetitive resistivity measurement processes have been reported on polymer materials, especially on Viton. The development of a reproducible resistivity test standard technique for the polymeric material such as Viton is of great interest.

Long operational hours at a high temperature constantly affect the polymeric materials. This is commonly known as thermal aging, which can drastically change the electrical properties and the chemical structure of the polymer. Exposed to the high-temperature environmental condition for long working hours, insulation polymers tend to lose their dielectric properties, leading to insulation failure [20,21,22]. It is necessary to find out the breakdown hours or insulation failure due to thermal aging by measuring the resistivity of polymeric materials.

When a large voltage is applied to a polymeric sample, the small amount of current flowing through the sample can be measured in order to obtain the resistance of the sample as a traditional accepted technique. This current value is extremely low. The challenging part of the testing protocol is measuring this small amount of current accurately and repeatably. Piezoelectric effects or discharging capacitive elements could be five to ten times the stimulated currents [23]. In the long-term period, the insulating material responds to the charge distribution, and the applied electric field may cause the change of resistivity of that material. Samples of the testing material’s temperature, humidity, initial voltage, etc. may cause variability in the results [24]. Therefore, an accurate testing protocol is needed to overcome this key challenge for the measurement of the resistivity of polymers.

In summary, during the operation of insulating the Viton fluoroelastomer in the high-temperature environment, the behavior of electrical properties such as volume/surface resistivity of the materials is still unclear. In order to ascertain the electrical properties of the insulating Viton, in this study, we investigated the effects of various parameters, e.g., transient effect, the temperature dependence of volume resistivity, and the voltage dependence of resistivity and surface morphology on the resistivity measurement using commercially available Viton specimens before and after the accelerated thermal aging process.

## 2. Background

A member of the FKM (*fluorocarbon*) family is known as the synthetic rubber Viton. The Viton polymer was first introduced by Du Pont in 1957. The American Society for Testing and Materials ASTM D1418 and the International Organization for Standardization ISO 1629 designation of FKM are categorized as Viton. Terpolymers of tetrafluoroethylene (TFE), hexafluoropropylene (HFP), perfluoromethyl vinyl ether (PMVE), as well as vinylidene fluoride (VDF) make up the Viton family. The commercially available standard Viton has three main cure chemistries of families. Vinylidene fluoride (VF2) and hexafluoropropylene (HFP) refer to the Viton family of A. Vinylidene fluoride (VF2), hexafluoropropylene (HFP), and tetrafluoroethylene (TFE) embraced with Viton family B and F. Due to the excellent resistance capability of low molecular weight esters, ketones, and aldehydes, Viton ETP was introduced in 1998, which was comprised of ethylene, tetrafluoroethylene (TFE), and perfluoromethyl vinyl ether (PMVE) [25,26,27,28,29]. Viton rubber has a density of over 1800 kg/m^3^, which is higher than other types of rubber polymer, and a fluorine content of 66 to 70% [30,31]. A high ratio of fluorine to hydrogen, strong carbon-fluorine bonds, and not having double bonds make the Viton polymer unique compared to other types of commercially available rubbers.

The chemical and thermal stability of elastomers increase due to the high bond strength. Due to its chemical formation, Viton has been shown to be resistant to mineral oil and grease, ASTM oil No. 1, IRM 902 and IRM 903 oils, silicone oil and grease, non-flammable hydraulic fluids (HFD), gasoline, chlorinated hydrocarbons (trichloroethylene and carbon tetrachloride), as well as aliphatic hydrocarbons (butane, propane, natural gas) [32].

Nowadays, polymers are attractive in industry applications for their superior electrical insulation properties. In general, since they have the capabilities to endure high electric voltages and are familiar as a good insulator for wires, polyethylene (PE), cross-linked PE, and polyvinyl chloride (PVC) are widely used in the industry. The research started several decades ago is still ongoing due to the high insulating polymer’s requirements in industry applications. At the same time, it is necessary to investigate the behavior of the existing polymers to identify their constraints during different operational conditions. Several efforts have been reported to the comprehension of the polymers’ insulation behavior and properties according to the measurement process. Dabbak et al. [33] carried out a study according to the ASTM D-149-97a standards to understand the temperature dependence of high voltage insulation breakdown of polyethylene/polypropylene compounds. Mohiuddin et al. [34] studied the temperature-and pressure-dependence of electrical resistance of (carbon nano tube) CNT-PEEK composites. Kato et al. [35] studied the uncertainty of electrical resistivity measurement in terms of film size, thickness, drying time, and electric field strength of biopolyimide poly(ATA-CBDA) and polyimide (Kapton). Thermal aging produces a large number of free radicals, a key factor for destroying the structure of polymers [36]. The authors of [37,38] investigated the thermal degradation of polymers, which affects the electrical insulation. The authors of [39,40] investigated the performance of the polymers for low and high voltages at different temperatures. For coatings in manufacturing industries, contact- electrode surface probes are the basic method of surface resistivity measurement [41]. For instance, using four-probe methods, Orlova et al. [42] measured the temperature dependence electrical resistivity and Hall constant R of the polymeric material. Moreover, they studied scanning and transmission electron microscopy (SEM and TEM) of medium-density fiberboards (MDF) materials. Similarly, Lukianova et al. [43] used a four-probe method to measure the electrical resistivity of materials. Naik et al. [44] measured the high-density polyethene (HDPE) material’s resistivity using a concentric ring probe. To measure the resistivity, Levy et al. [45,46] and Frederickson et al. [47,48] developed a charge storage method, and Fernando et al. [49] introduced a non-Gaussian approach to determine the confidence intervals for the electrical surface resistivity of polymers. However, the behavior of the polymers’ electrical properties during an operation is still unclear, which restricts their applications in the industry. Therefore, extensive research should be carried out on different electrical resistivity measurement techniques to better understand the cause of insulation failure of polymeric materials under the operational environmental conditions.

## 3. Experimental Process

### 3.1. Materials

The polymer employed in this experimental study is the chemical-resistant Viton Fluoroelastomer Rubber (FKM), supplied by the USA company McMASTER-CARR. The material has the useable outdoor capability with a temperature range of −29 to 204 °C, tensile strength of 1000 psi, durometer 75A (hard) with tolerance −5 to +5, black in color, RoHS 3 (2015/863/EU) compliant, and REACH (EC 1907/2006) (06/25/2020, 209 SVHC) compliant [50]. The components used in this study are all commercial products and, to improve reliability, all the samples were cut from the same batch of materials. The experiments were conducted at the “Product and Process Design Lab” of the University of Oklahoma, Norman, USA. Table 1 reflects the parameters of the sample.

### 3.2. Test Apparatus

The resistivity measurement was conducted by several instruments: A two-probe setup (TPX-200 C) and a 6517b Keithley electrometer connected to an 8009 Keithley test fixture.

#### 3.2.1. Two-Probe Method (TPX-200 C)

**Figure 1:**
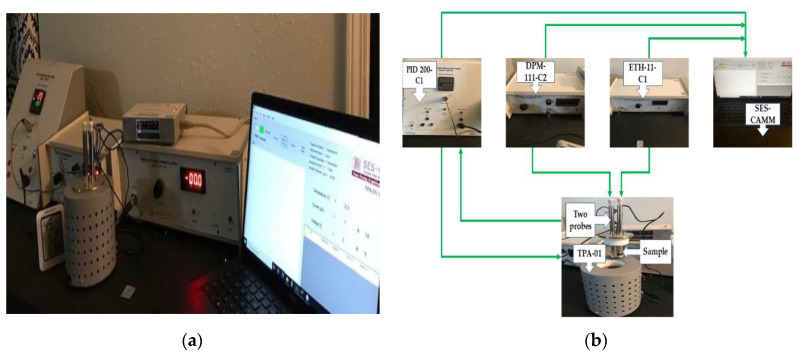
Resistivity measurement setup 1 (TPX-200 C). (**a**) Overall setup, (**b**) block diagram of connections.

TPX-200 C is a well-known and commonly used two-probe fixture of the high resistivity samples for the resistivity measurement, which is beyond the range of the four-probe method. The experimental setup includes: (1) A two-probe arrangement, e.g., TPA-01 containing two spring-load contact probes insulated with Teflon, able to move in a pipe, mounted in a stand, sample plate, and RTD sensor; (2) a PID controlled oven (PID-200-C1); (3) a digital Pico ammeter (DPM—111-C2) with the minimum current measurement of 10^12^ A; (4) a high voltage power supply with a maximum of 1500 V output (EHT-11-C1); and (5) a computer interface (SES-CAMM) for data acquisition (current, temperature, and voltage) [51]. Figure 1 illustrates the overall setup of the TPX-200 C and the connection block diagram of the setup components.

Volume resistivity represents a polymeric material’s strength to oppose the flow of leakage current through the material’s cubic specimen body. Resistivity *ρ* (Ohm-m or Ohm-cm) is calculated by the following Equation (1):

(1) $$\rho =\frac{R\cdot A}{L}$$

where *R* is the resistance of a specimen, *A* is the area, and *L* is the thickness. The generalized Ohm’s law can also represent volume resistivity by means of the electric field *E* and current density *J* using the following Equation (2):

*E* = *ρJ* (2)

Resistivity can also relate two vector quantities as the second rank of tensor according to the following Formula (3):

*E_i_* = *ρ_ik_J_k_* (3)

The tensors may represent with 3 × 3 matrices and the vectors with 3 × 1 matrices. This is a complex way to resistivity measurement and is used in anisotropic cases, where anisotropic means polymers with different properties in different directions. Alternatively, the reciprocal of the resistivity is known as conductivity σ with the unit of (Ω^−1^ m^−1^ or Ω^−1^ cm^−1^). The value range < 10^3^ (Ω m) represents conductors; the range 10^3^–10^9^ (Ω m) represents antistatic materials; and the range > 10^9^ (Ω m) is known as insulators [8].

With the help of the 2D Laplace equation solution, the resistance between two probes/electrodes touching a conductive surface of the material can be determined. The circular electrodes distance is noted as *d* and the radius of the electrodes is noted as *r*. Theoretically, the resistance behaves independently of the electrode’s separation, e.g., *d >> r.* Furthermore, the theory describes that most of the voltage drop happens at the immediate vicinity of the electrode’s tip. The two-probe/electrode method has the ability to measure the polymer’s resistivity in the small area around the probe’s tip where, theoretically, the voltage drop happens. Sometimes, due to the high current density around the probe/electrode’s tip, the polymer sample overheats. A disc-shaped probe/electrode tip is recommended for this method, which can exert some light pressure on the sample surface in order to minimize the material distortion and to define a more precise area of contact. Therefore, the contact between the electrode and sample will improve, and the distortion of the sample material will decrease [8]. Here, from Figure 1b, it can be seen that TPA-01 has a disc-shaped probe tip, which is recommended for the resistivity measurement. A PID-controlled oven (PID-200-C1) is connected to the probes to control the high temperature range accurately, a digital Pico ammeter (DPM—111-C2) measures the current reading, and a high voltage power supply (EHT-11-C1) has been used to supply the voltage in the desired range. Finally, the computer interface (SES-CAMM) records all the data for further analysis.

#### 3.2.2. Alternating Polarity Method (Keithley 6517b + 8009)

To improve the resistivity measurements of polymers, the alternating polarity method was developed. The electrostatic discharge (ESD) phenomenon happens when electric charges are stored on material surfaces. In industrial applications, the electrical resistivity and characteristics of the materials are the factors on which the level of the electrostatic discharging depends. When the level of electrostatic charges increases incrementally, then the surface discharges occur. The antistatic or static dissipative is a key factor for insulating materials of the electrostatic charge accumulation phenomenon. The electrostatic charge accumulation is a key issue for the insulating materials’ electrical properties such as resistivity measurement [52]. In order to overcome this problem, an advanced resistivity measurement approach is needed which has the ability to measure the polarization current. The alternating polarity method, which is the recommended method for materials with high resistivity, was used in this setup. This setup has been used to measure the resistivities at room temperature due to its superior accuracy and resolution. Background currents which may develop from many sources, e.g., piezoelectric effects, temperature, or triboelectric effects, can affect the measurement accuracy and increase the error. One common solution to reduce the effects of background current is using the alternating polarity method in which the voltage cycles between positive and negative values. A positive voltage (V) and a negative voltage apply as a cycle for recording the currents (*I*_1_, *I*_2_, *I*_3_, *I*_4_, *I*_5_) [53,54,55]. The weighted averaged current calculation formula is Equation (4):

(4) $$I_{\mathrm{avg}}=\frac{-I_{2}+3I_{3}-3I_{4}+I_{5}}{8}$$

A significant transient current (*I*_1_) would be initiated in the first cycle. To improve the measurement accuracy, the first cycle should be excluded from the calculation [53]. The effective area *A* of the guarded electrode can be obtained by the following Formula (5):

(5) $$A=\pi \frac{{\left(d_{1}+g\right)}^{2}}{4}$$

where *g* is the distance between the guarded electrode and the ring electrode, and *d_1_* is the diameter of the guarded electrode. Finally, having the value of area A, thickness t, average current *I*_avg_, and alternating voltage V with the help of Equation (6) below, one can calculate the volume resistivity *ρ* [53,55].

(6) $$\rho =\frac{V\cdot A}{I_{\mathrm{avg}}\cdot t}$$

**Figure 2:**
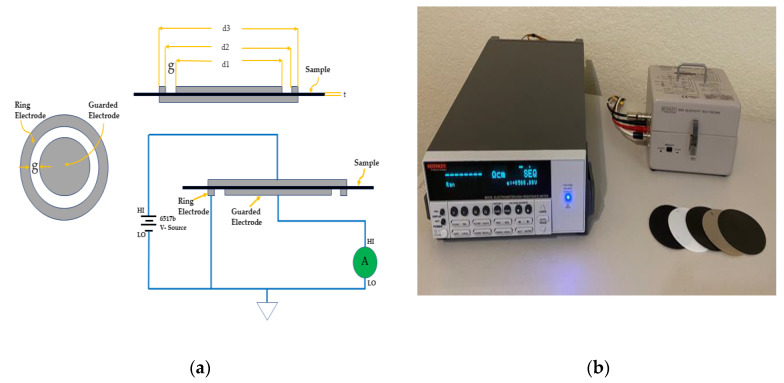
(**a**) Schematic of a typical electrometer resistivity test; (**b**) resistivity measurement setup 2 (Keithley 6517b + 8009).

Figure 2a demonstrates a schematic of a typical resistivity measurement test fixture, and Figure 2b illustrates the resistivity measurement setup 2 (Keithley 6517b + 8009) employed in this study. This setup is our preferred method of measuring the resistivity whenever we do not need to increase the temperature, and also has been used to validate the measurements of TPX-200 C.

### 3.3. Scanning Electron Microscopy (SEM)

A Hitachi TM3000 scanning electron microscope (SEM) (Hitachi High-Tech Co., LTD, Tokyo, Japan) was used in this study to investigate the surface morphology of specimens before and after the accelerated aging process [56]. With a 30 nm resolution, a charge reduction mode of 5 or 15 kV voltage, and ×15 to ×30,000 magnification capabilities, the Hitachi TM 3000 SEM is a good fit for closely observing the surface of the specimens. A height adjustment screw was used to adjust the height of the specimen, and no coating was applied since the charge indemnification was employed to dissipate the charge accumulation. The SEM images were taken on 500, 300, and 200 μm focal lengths. This part of the experiment was conducted at the “Samuel Roberts Noble Microscopy Laboratory“ of the University of Oklahoma, Norman, USA.

### 3.4. Aging Process

To investigate the aging effects of Viton specimens, the aging process was conducted in this study. A constant temperature oven was used for the thermal aging of this experiment. The temperature was selected at 225 °C. The Viton samples were submerged in water inside an aging cylinder in the oven to degrade the material’s structure. The aging process lasted nearly 4 days or 100 h. All the Viton samples were identified and numbered before and after aging.

After the process, the aged samples were kept in a dehumidifying chamber for 2 weeks. The weight of the samples was measured before the aging process and after the 2-week wait to detect any possible water absorption during the aging process. Following these steps, all the tested samples showed no difference in their weight indicating a negligible water absorption.

### 3.5. Resistivity Measurement

Resistivity, which is then a normalized version of resistance, is independent of the size of the material and can be considered a material property rather than a specimen property. Resistivity generally is expressed in two ways: Volume resistivity and surface resistivity.

The experiment maintained a single protocol developed by the team for resistivity measurement. A commercially available dehumidifier chamber (Bel-Art^TM^ Secador^TM^ Auto Desiccator Cabinets: Vertical Models) [57] was used to make sure all the samples are moisture-free. The resistivity measurement was conducted by the TPX-200 C with the following steps and protocols:

- Cleaning the electrodes and samples with alcohol wipes frequently to avoid any contamination.
- Each sample rested for at least 48 h after each test before being tested again in order to remove any stored charge inside the sample. This is due to the fact the insulating materials behave similarly to the capacitors and store charges inside them. To have an accurate measurement, any charge history needs to be removed.
- Right before applying the voltage, any current reading was zeroed to prevent an inaccurate measurement. Especially for measurements at high temperatures, this is necessary since the temperature variation by itself creates a current inside the specimen.
- Sixty seconds of electrification as suggested by ASTM D257 [55].
- Readings were recorded 60 s after applying the voltage to the specimen.

The resistivity measurement was conducted by the alternating polarity method (Keithley 6517b + 8009 resistivity fixture) with the following steps and protocols:

- Cleaning the electrodes and samples with an alcohol wipe frequently to avoid any contamination.
- Similar to the two-probe method, each sample rested for at least 48 h after each test in order to be tested again to remove any stored charge inside the sample.
- Each alternation lasted for 60 s.
- At least 10 alterations were performed to stabilize the reading.
- For the alternating polarity test, there were four alternations before the first reading to calculate the average current.

## 4. Results and Discussion

### 4.1. The Transient Effect

Prior to investigating how the resistivity of Viton is changing while altering various parameters, it is necessary to understand the mechanism of conductivity in insulators. In polymeric insulators with high resistivity, the charged species and polarization of dipole moments are mainly responsible for the electrical conduction [53].

Upon applying a potential difference to the insulator, two types of charging currents are created. One type is instantaneous, which results from the electronic and atomic polarizations. The second type develops slowly due to the dipole and interfacial polarizations [58]. The latter has a substantial relaxation time, which is defined as the time it takes for the polarizations to disappear or form. This long transient time can affect the accuracy of resistivity measurements.

Two typical methods to reduce the transient effects are waiting for an acceptable rate of resistivity change and the alternating polarity method. The main difficulty of the first approach is that it takes a long time to reach a steady state, if it happens at all. The alternating polarity test will solve this problem by repeatedly reversing the charge direction in order to achieve a reproducible transient measurement. The American Society for Testing and Materials (ASTM) suggests intervals of 60 s with ±500 V of an electrical source [55].

When the external electric field is removed, the internal electric field will dictate the direction in which the charge carriers can move, resulting in a reverse current inside the specimen [59,60]. Due to the large size of some of the charged species and their slow movement, the reverse current might be present for a long time after removing the external electric field. This reverse current is also present during the current/resistivity measurements resulting in a constantly decreasing current until reaching a stable condition. This phenomenon is known to be due to the dielectric absorption and sweep of mobile ions to the electrodes. The decrease in the current with time can be shown in the form of *I(t) = At^−m^*, where A is a constant and m is usually between zero and one [61]. Therefore, the resistivity keeps increasing after applying the electrical field to the specimen until reaching stable conditions, which might take hours or days [53]. Since it is not feasible to wait that long for each test, it is recommended to wait about 2 min before measuring the resistivity [55].

**Figure 3:**
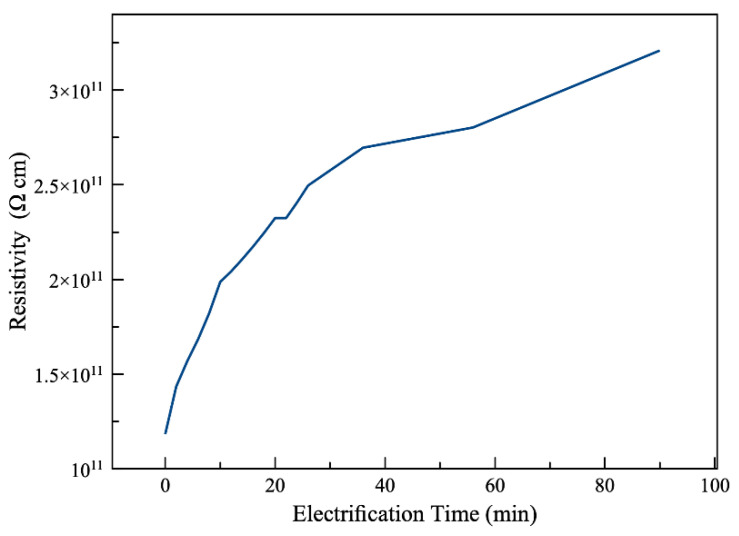
Volume resistivity with respect to time of a Viton sample under constant 500 V DC (less than 1% error).

Our data show the rate at which the current is decreasing after applying the voltage, which is very high for the first 60 s and gradually decreasing afterwards. Figure 3 shows the volume resistivity of Viton with respect to time after applying the electric source (electrification time) under 500 V. There is a 21% increase in resistivity in the first 2 min, which is by far the greatest surge throughout the whole graph. Our finding then supports the necessity of waiting for 2 min before taking the measurements. While this graph reports the data up to 90 min, the resistivity keeps increasing for a much longer time as reported in the literature [53].

### 4.2. Temperature Dependence of Volume Resistivity

**Figure 4:**
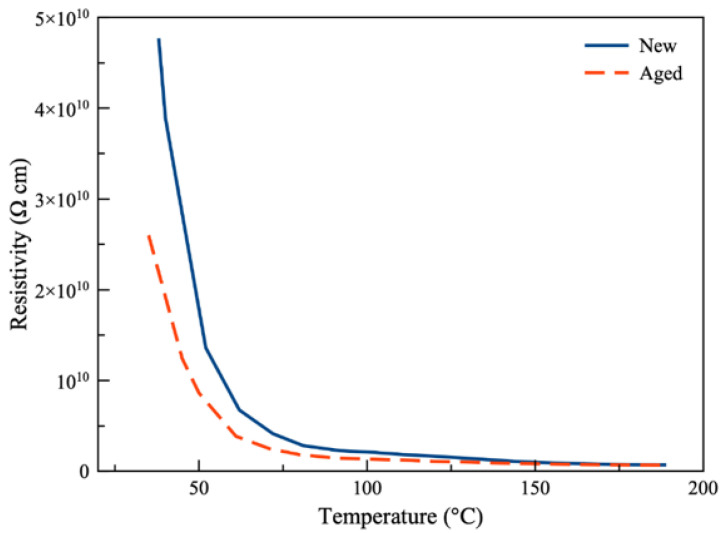
Volume resistivity of Viton specimen at various temperatures ranging from 35 to 190 °C at 500 V (less than 2% error).

The temperature dependence of resistivity is one of the main objectives of this study. The TPX-200 C setup provides the flexibility of measuring the volume resistivity up to 200 °C. Since this setup is not capable of performing the alternating polarity test, the standard test procedure with ASTM recommendations is employed. Figure 4 shows the volume resistivity of Viton specimen at various temperatures ranging from 35 to 190 °C at 500 V, as recommended for the insulating materials of the new and aged samples. As expected, for both samples, the volume resistivity decreases with the increasing temperature, indicating the negative coefficient of resistance.

The mechanism behind this decrease is not fully understood yet since it is not feasible to observe this phenomenon closely. However, several studies reported a similar trend of lower resistivity at higher temperatures and related this decrease to the re-agglomeration of carbon black aggregates to be responsible for the observed decline in the resistivity [62,63]. Additionally, the increase of the free volume size inside the polymers due to the temperature rise has been shown to increase the conductivity, which is another possible explanation for the observed decrease in resistivity [64].

At lower temperatures, the plots representing the resistivity of the new and aged samples are more divergent. However, the separation is constantly decreasing, and the two plots are converging as the temperature is rising. That is, the resistivity of the new sample is decreasing at a higher rate compared to the aged sample. At around 35 °C, the resistivity of the aged sample is shown to be around 45% lower than the new sample, while this difference becomes 15% at 150 °C. The resistivity readings for both samples are very close after 150 °C. A temperature surge from 35 to 190 °C leads to the resistivity decreasing from 4.77 × 10^10^ to 6.99 × 10^8^ Ω cm for the new sample and from 2.6 × 10^10^ to 6.68 × 10^8^ Ω cm for the aged sample.

The material degradation resulting from the aging process led to a decrease in the ability of the polymer insulator to resist the external electric field. It is well expected since the insulating materials generally lose their dielectric characteristics after being exposed to environments with high temperature and humidity for a considerable amount of time. The accelerated aging process used in this study is not significantly vigorous, but still the impact is noticeable. After investigating the surface morphology, an explanation will be provided for the different rates at which the resistivity is decreasing for the new and aged samples.

### 4.3. Voltage Dependence of Resistivity

A wide variety of materials obey Ohm’s law, in which the voltage and current are to be directly proportional to each other. Therefore, the resistance or resistivity remains constant even if the voltage and current are changing. These materials (such as resistors and capacitors) are called ohmic materials. For ohmic materials, the voltage-current curve is linear with a constant slope, which is the resistance. On the other hand, there are materials that do not follow Ohm’s law and are called non-ohmic materials. In the case of non-ohmic materials (such as diodes and transistors), the voltage-current curve is not linear, leading the slope (resistance) to be varying at any point.

Insulating materials usually behave as non-ohmic materials, which is why the voltage used to measure the resistivity is always necessary to be reported [55]. As mentioned earlier in this article, we followed the recommendation of ASTM and measured the resistivity under a voltage of 500 V. However, it can be beneficial to observe how voltage-sensitive the resistivity is.

**Figure 5:**
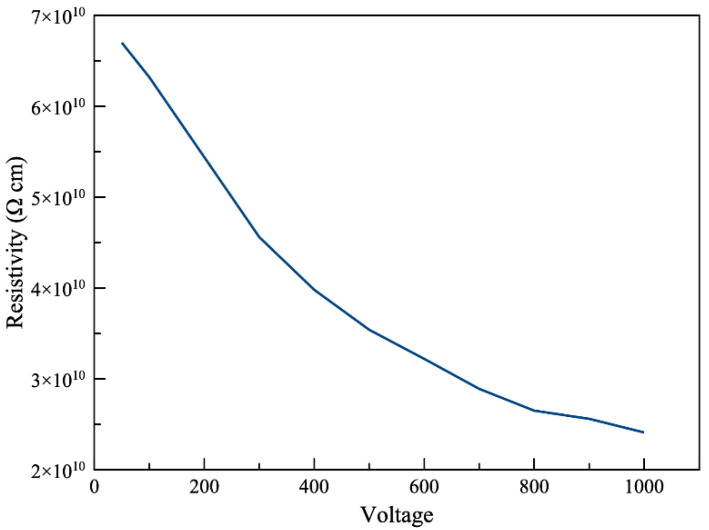
Volume resistivity of Viton specimen under various voltages (less than 1% error).

Figure 5 shows the relationship between the voltage and resistivity of a new Viton sample. It is clear that as the test voltage increased, the measured resistivity decreased. A voltage surge from 100 to 1000 V resulted in resistivity to drop more than half. This curve can be very helpful in selecting the best testing voltage based on the application of the specimen. For example, if the specimen is designed to undergo very high voltages, high test voltages can produce more accurate measurements for the real application of the material. Several studies also reported similar resistivity-voltage curves, in which the resistivity is decreasing with the increasing voltage [65,66,67].

### 4.4. Surface Morphology

The surface morphology is one of the key factors affecting the performance of insulators. The impact of aging on the surface morphology is more significant in the case of composite insulators compared to glass and porcelain insulators [68]. Therefore, the surface morphology of the new and aged samples is investigated in this study through scanning electron microscopy (SEM) to closely observe the impact of aging.

**Figure 6:**
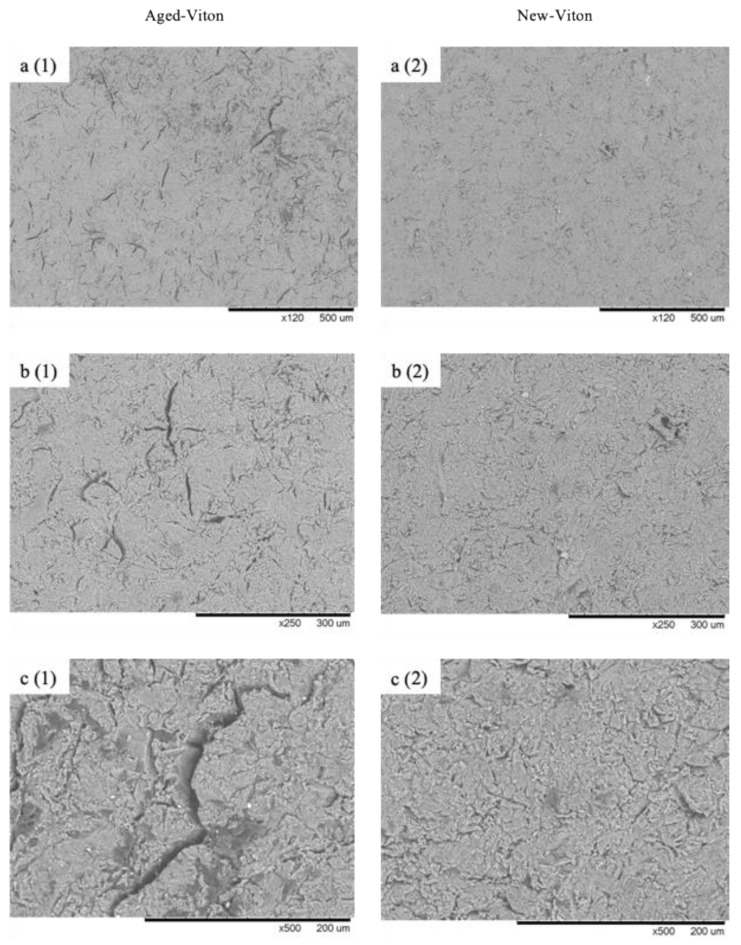
SEM micrographs of the new and aged Viton sample. (**a**) ×120, (**b**) ×250, (**c**) ×500 magnification.

Figure 6 provides several SEM images taken side by side from the surface of our new and aged Viton samples. The relatively low magnification (×120) in Figure 6(a1,a2) represent an area of more than 1 mm^2^ of the samples, which is desirable to observe the overall surface morphology. It can be clearly seen that the surface of the new sample is much smoother with fewer and smaller imperfections compared to the aged one. The accelerated aging process notably increased the surface roughness and the severity of the cracks.

The higher magnification images in Figure 6(b1,b2,c1,c2) are more suitable to investigate how cracks and surface imperfections are affected by aging. A higher number of cracks with significantly larger dimensions is evident from the images of the aged sample (b1, c1) compared to that from the new sample (b2, c2).

The aging process clearly created more cracks and exacerbated the existing cracks. Deep and huge cracks are only present on the surface of the aged sample. The image contrast can be used to estimate the severity of the cracks. The majority of cracks on the aged sample (a1, b1, c1) show a considerably higher contrast (the difference between the darkest and lightest gray shade) in comparison with the new sample (a2, b2, c2). That is, the darker the cracks, the deeper they are. All imaging parameters (such as brightness and contrast) were kept constant when the SEM images were taken. Both samples were inserted together inside the microscope to make sure all the microscope parameters were identical for both. The size of the cracks can be easily estimated via the scale bar below each image. Many elongated cracks with more than 200 μm in length can be found on the surface of the aged sample (a1, b1). One of these huge cracks is shown in Figure 6(c1) with a length of around 300 μm, as an example.

The presence of cracks on the surface of insulators is recognized to have a negative effect on their resistivity and dielectric properties. The cracks are known to accumulate pollutants and contaminations (such as carbon) from the environment, forming conductive deposits [69]. Additionally, the cracks are prone to absorb moisture, which can create a conductive film and decrease the ability of the insulator to prevent electrical discharge efficiently.

The presence of large cracks on the aged sample indeed decreased the resistivity, as shown in Figure 6. One interesting observation from Figure 4 was the fact that as the temperature increased, the resistivity of the aged sample grew closer to that of the new sample. The SEM images make it possible to propose a possible explanation for this observation. As the temperature increases, more moisture is evaporated and, thus, the cracks contain less moisture and the contaminations and resistivity grow closer to the new sample. Especially after passing 100 °C, the difference becomes significantly smaller since the moisture and contaminations will evaporate at a higher rate.

Another factor can be the time. The whole test from 35 to 190 °C usually takes about 90 min and about 60 min from 100 to 190 °C. Therefore, as the experiment continues and the temperature increases, the moisture and contaminations are exposed to the high temperature for a longer time, which can affect the evaporation. Last but not least, the physical characteristics of the insulator will alter at higher temperatures. The elevated temperature will soften the material along with a possible expansion. These physical changes might affect the size and shape of the cracks in a way that the resistivity decreases at a lower rate compared to the new sample.

## 5. Conclusions

In this study, the electrical insulation property of volume resistivity for new and aged Viton samples was presented. It was clear from the graphs that the electric resistivity of the aged Viton sample was found to be lower compared to the new Viton sample. In the process of resistivity measurement, specifically for the first 60 s after applying the voltage, the resistivity increased due to a high rate of the current drop. To be more specific, around 21% increase in resistivity was observed in the first 2 min under 500 V, which supports the necessity of a 2-min waiting before recording the measurement, as suggested by ASTM D257. The volume resistivity of Viton strongly depended on the temperature, voltage, electrification time, and charge accumulation occurrence. The applied voltage has been shown to be very crucial to obtain an accurate resistivity measurement since Viton behaves similar to a non-ohmic material. The aged Viton sample showed a much lower value of volume resistivity compared to the new Viton sample until 100 °C. However, as the temperature increases, the resistivity of the aged sample grows closer to the new sample. For example, at around 35 °C, the resistivity of the aged sample is shown to be around 45% lower than the new sample, while this difference becomes 15% at 150 °C under 500 V. The SEM images allowed us to successfully explain this observation. The surface of the new sample is much smoother with fewer and smaller imperfections compared to the aged one, and the aging process notably increased the surface roughness and the severity of the cracks for the aged samples. The large cracks on the surface of the aged sample allow contamination and moisture to be trapped and consequently, decrease the resistivity. However, when the sample is exposed to high temperatures, the trapped moisture and contamination will be partially evaporated, leading to a closer resistivity to the new sample. The future extension of this work will possibly explore the ultraviolet (UV) radiation aging effects on a Viton sample in different operational conditions for a better understanding of the electrical insulation characteristics. Analyzing the crystalline structure of the new and aged samples can be beneficial. A higher resolution SEM microscope or a TEM microscope with careful sample preparation will be employed in order to analyze the internal structure of the samples further, along with more details in a future extension of this work.

## Figures and Tables

**Table 1:** Parameters of the polymer (Viton) sample used in the experiment.

Material	Thickness (mm)	Area (mm2)	Setup	Sample Size
Viton (McMaster-Carr)	Thin (0.7493 ± 0.0127)	113.097	(TPX 200C)	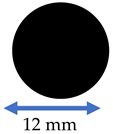
	Thick (1.47066 ± 0.0127)	147.3	(Keithley 6517b + 8009)	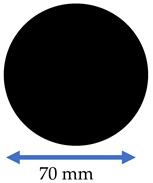

## Data Availability

Not applicable.
